# Endocrine Factors Modulating Immune Responses in Pregnancy

**DOI:** 10.3389/fimmu.2014.00196

**Published:** 2014-05-08

**Authors:** Anne Schumacher, Serban-Dan Costa, Ana Claudia Zenclussen

**Affiliations:** ^1^Department of Experimental Obstetrics and Gynecology, Medical Faculty, Otto-von-Guericke University, Magdeburg, Germany; ^2^University Women’s Clinic, Otto-von-Guericke University, Magdeburg, Germany

**Keywords:** progesterone, estradiol, human chorionic gonadotropin, luteinizing hormone, alpha-fetoprotein, immune regulation, pregnancy

## Abstract

How the semi-allogeneic fetus is tolerated by the maternal immune system remains a fascinating phenomenon. Despite extensive research activity in this field, the mechanisms underlying fetal tolerance are still not well understood. However, there are growing evidences that immune–immune interactions as well as immune–endocrine interactions build up a complex network of immune regulation that ensures fetal survival within the maternal uterus. In the present review, we aim to summarize emerging research data from our and other laboratories on immune modulating properties of pregnancy hormones with a special focus on progesterone, estradiol, and human chorionic gonadotropin. These pregnancy hormones are critically involved in the successful establishment, maintenance, and termination of pregnancy. They suppress detrimental maternal alloresponses while promoting tolerance pathways. This includes the reduction of the antigen-presenting capacity of dendritic cells (DCs), monocytes, and macrophages as well as the blockage of natural killer cells, T and B cells. Pregnancy hormones also support the proliferation of pregnancy supporting uterine killer cells, retain tolerogenic DCs, and efficiently induce regulatory T (Treg) cells. Furthermore, they are involved in the recruitment of mast cells and Treg cells into the fetal–maternal interface contributing to a local accumulation of pregnancy-protective cells. These findings highlight the importance of endocrine factors for the tolerance induction during pregnancy and encourage further research in the field.

## Introduction

It is said that mammalian pregnancy defies the immunological rules because a semi-allogeneic conceptus is tolerated rather than rejected. It is therefore, a fascinating phenomenon and target of many immunological studies. Pregnancy is however a natural phenomenon that ensures the survival of species and exists since millions of years. By contrast transplantation, where empirical observations led to the definition of the immunological rules, is an artificial process that was first described in 1905. Thus, understanding how natural tolerance operates may help creating novel strategies to ensure tolerance in other models. Especially because of the fact that initial allorecognition of foreign fetal antigens by the maternal immune system is advantageous for a successful pregnancy, the mechanisms behind gestational tolerance are of interest for other disciplines. Local suppression of alloreactive immune responses to paternal antigens is a prerequisite for fetal acceptance. Steroid hormones like progesterone (P4) and estradiol (E2) as well as gonadotropins such as the human chorionic gonadotropin (hCG) are fundamentally involved in the regulation of the menstrual cycle and in the establishment and maintenance of pregnancy (1, 2). Through binding their specific receptors expressed by immune cells and/or by acting via mediators these hormones support fetal tolerance by inhibiting destructive immune responses and inducing tolerance pathways. This review highlights the effects of pregnancy-associated hormones on different immune cell types with a special focus on P4, E2, and hCG.

## Progesterone

P4 is a member of the steroid hormone family and has been described as the “pregnancy hormone” due to its indispensable role for pregnancy maintenance (3). During the menstrual cycle, P4 levels are relatively low during the preovulatory phase, rise after ovulation, and are elevated during the luteal phase (4). If pregnancy occurs, hCG initially maintains P4 levels by inducing its production by the *corpus luteum*. After the luteal–placental shift, the placenta takes over P4 production (5). P4 prepares the uterus for implantation as it induces differentiation of stromal cells into decidual cells (decidualization) and decreases the contractility of uterine smooth muscle cells (6, 7). Additionally, P4 withdrawal is associated with the initiation of labor (8). P4 has been shown to affect immunity, mainly at pregnancy concentrations. These effects are primarily mediated via the intracellular P4 receptors (PR), PR-A and PR-B, which act as transcription factors, although non-genomic effects of PR activation have been reported (9). In addition, P4 mediates its immune regulatory function via mediators such as the progesterone-induced blocking factor (PIBF) and glycodelin A (10, 11).

## Estradiol

Like progesterone, estrogens belong to the steroid hormones. Three major naturally occurring estrogens have been described in women, namely estrone (E1), estradiol (E2), and estriol (E3). Within those, E2 is the predominant estrogen produced during the reproductive years. High levels of E2 are produced by the ovary, while smaller amounts are also produced by the adrenal cortex and from E2 precursors in fatty tissues (12). In the normal menstrual cycle, E2 levels rise with follicular development, drop briefly at ovulation, and rise again during the luteal phase for a second peak. At the end of the luteal phase, E2 levels drop to their menstrual levels unless there is a pregnancy (13). During pregnancy, E2 levels increase continuously until term due to the production by the growing placenta (14). Several important functions have been described for E2. During the menstrual cycle, E2 triggers the luteinizing hormone (LH) surge resulting in ovulation. After ovulation, in the luteal phase, E2, in conjunction with P4 prepares the endometrium for implantation. Upon pregnancy, E2 is shown to promote uterine blood flow, myometrial growth, stimulate breast growth and at term, promote cervical softening and expression of myometrial receptors. Besides, E2 was suggested to affect different immune cell populations in their number and function and thereby contributes to fetal tolerance. These effects are mediated via binding of E2 to its intracellular receptors, estrogen receptor alpha (ERα) and beta (ERβ), which in turn modulate the expression of many genes (15). Both receptors are expressed in various lymphoid tissue cells as well as in lymphocytes, macrophages, and dendritic cells (DCs) (16, 17).

## Human Chorionic Gonadotropin

Human chorionic gonadotropin is a primate-specific heterodimeric placental glycoprotein. Four different hCG variants, namely total hCG, hyperglycosylated hCG (hCG-H), free β-subunit, and pituitary hCG, have been reported, each produced by different cells with separate biological functions (18). In humans, after pregnancy onset, total hCG increases rapidly during the first trimester, peaks between the 9th and 12th week of pregnancy and then declines, until the woman gives birth, although remaining higher than in a non-pregnant woman (19). hCG is produced by differentiated syncytiotrophoblasts and its main function is to stimulate P4 production by the *corpus luteum* (20). Moreover, hCG supports pregnancy by facilitating trophoblast invasion (21–23), promoting angiogenesis, and ensuring nourishment of the fetus (24–26). In rodents, similar functions are mediated by the highly homologous LH. During the last years, there is growing evidence that hCG and LH are involved in immune tolerance mechanisms leading to fetal survival. Both gonadotropins were shown to affect immune cells by binding to the LH/CG receptor expressed by several immune cell types. Moreover, hCG also acts through the mannose receptor.

## Alpha-Fetoprotein

Alpha-fetoprotein (AFP) is a glycoprotein that is produced by the yolk sac and fetal liver during pregnancy (27). It is the most abundant plasma protein found in the human fetus, acting as a fetal transport protein. AFP levels increase in the 4-week-old fetus, peak between the 12th and 16th week and remain low after birth. Although several studies provide evidence for an immune regulatory potential of AFP (28–32), it is still not explored whether AFP contributes to pregnancy success by modulating immune responses.

## Hormonal Influence on Immune Cells during Pregnancy

### Effect of pregnancy hormones on macrophages

**Figure 1:**
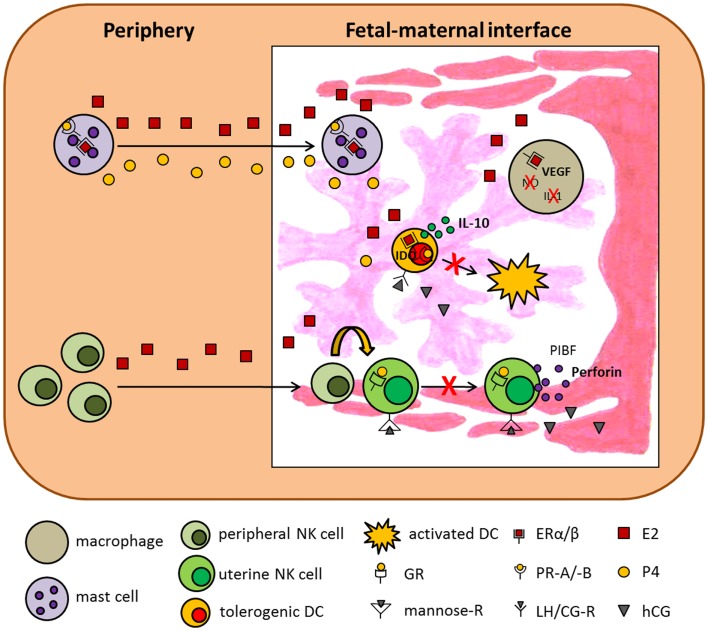
**Hypothetical scenario presenting the influence of pregnancy-associated hormones on innate immunity**. The scenario suggests several mechanisms by which E2, P4, and hCG influence innate immune cells and thereby support pregnancy success. ER, estrogen receptor; GR, glucocorticoid receptor; IDO, indoleamine 2,3-dioxygenase; IL-1, interleukin-1; IL-10, interleukin-10; LH/CG-R, luteinizing hormone/chorionic gonadotropin receptor; NO, nitric oxide; PIBF, progesterone-induced blocking factor; PR, progesterone receptor; VEGF, vascular endothelial growth factor.

Monocytes and macrophages are major representatives of the innate immune system in the cycling and pregnant mammalian uterus. Several studies provide evidence that monocyte recruitment, differentiation into macrophages, and function in the reproductive tract is modulated by pregnancy-associated hormones (33). Hormonal influence may be achieved by directly binding to the appropriate hormone receptors expressed on human and murine macrophages (16, 34, 35) or indirectly by modulating the levels of cytokines and growth factors that target the resident macrophages and influence their secretory profile. Hunt and colleagues reported that P4 reduced macrophage migration into the murine uterus (36), while Kitzmiller and colleagues showed that E2, P4, and hCG did not affect macrophage migration in guinea pigs (37). Differentiation of monocytes into macrophages was hindered by glycodelin A, a P4 mediator, by induction of apoptosis in human monocytes. However, after differentiation glycodelin A was not able to alter phagocytic capacity of macrophages (11). Macrophages are important regulators of trophoblast activity that promote tissue remodeling and angiogenesis (38). In this regard, E2, hCG, and LH have been demonstrated to enhance the production of the vascular endothelial growth factor (VEGF) in human macrophages (39, 40), supporting vessel formation in the placenta. In addition, P4 impairs the ability of human and murine macrophages to produce potent effector molecules such as nitric oxide and IL-1 proven to be detrimental for successful pregnancy outcome (36, 41, 42). Moreover, P4 suppresses toll-like receptor-triggered activation of murine macrophages by regulating miR-155 expression (43). Menzies and colleagues recently suggested an involvement of P4 in the regulation of genes associated with alternative macrophage activation (44). By contrast, hCG treatment of human and murine IFN-γ-primed macrophages resulted in increased production of nitric oxide, reactive oxygen species, IL-6 and IL-12p40, and enhanced phagocytosis of apoptotic cells (45, 46). However, hCG treatment of murine IFN-γ-primed macrophages did not affect the induction of allogeneic T cell proliferation (45). Interestingly, macrophages regulate excess of hCG known to be teratogenic to fetal tissues. Here, human macrophages are proposed to incorporate and degrade hCG in a time-dependent manner that protect fetal gonadogenesis from excess hCG (47, 48). More precisely, Katabuchi and colleagues recently demonstrated that hCG induces transient vacuole formation in human monocytes, morphologically mimicking Hofbauer cells. The authors suggest that Hofbauer cells and especially their vacuoles are involved in the protection of fetal tissue from high amounts of maternal hCG (49). Besides an effect of steroid hormones and gonadotropins on monocytes and macrophages, AFP is suggested to have an influence on both innate immune cell types. It has been demonstrated that AFP significantly suppresses the production of TNFα and IL-1β and induces a rapid down-regulation of surface MHC class II expression in a stimulated human monocyte cell line (29, 31). Moreover, Lu and colleagues showed that AFP inhibits the cell surface expression of Ia antigens on macrophages but does not affect macrophage viability (50). Hormonal effects on macrophages are summarized in Figure 1.

### Effect of pregnancy hormones on natural killer cells

**Figure 2:**
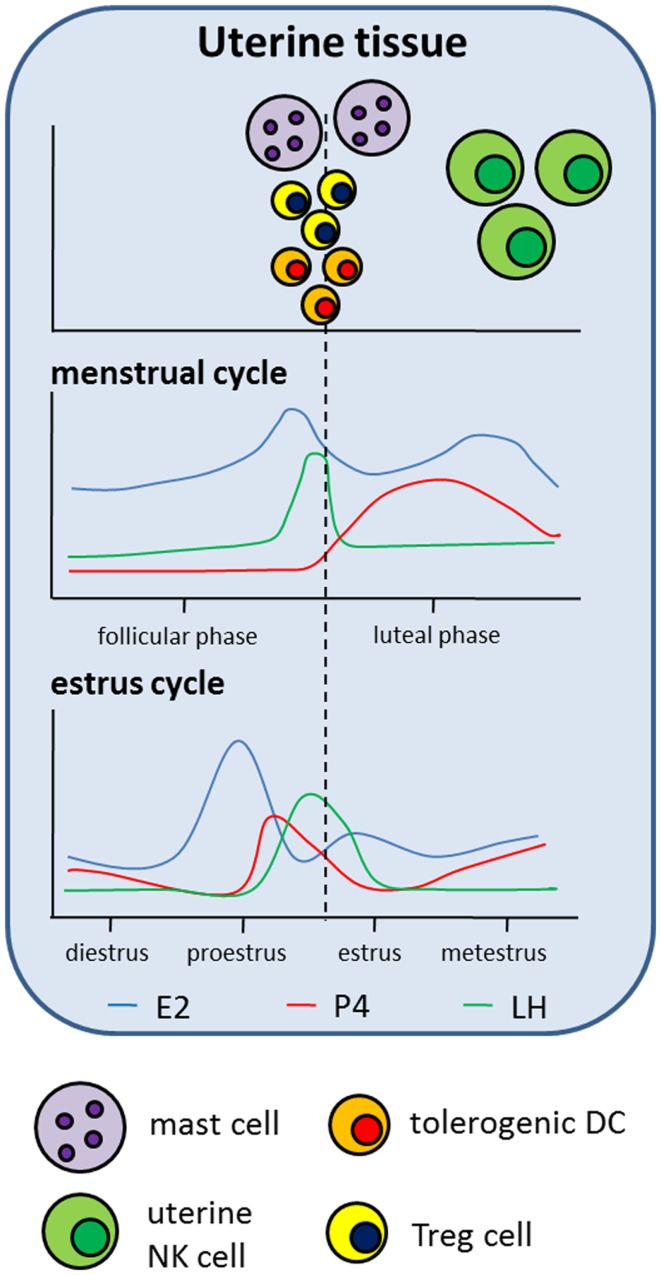
**Hypothetical scenario presenting the influence of hormonal changes taking place during the reproductive cycle on the distribution of innate and adaptive immune cell populations in the uterus**. The upper part of the scenario displays the accumulation of different immune cell populations in uterine tissue correlated with the different phases of the human menstrual cycle and the murine estrus cycle. The lower part of the scenario displays hormonal changes taking place during the reproductive cycles, both in humans and mice.

NK cells and, in particular, uterine NK (uNK) cells are of special interest when analyzing mechanisms underlying normal pregnancy. This becomes obvious when taking into account that uNK cells are the predominant lymphocyte population in the late secretory phase of the menstrual cycle and in the early pregnant uterus representing circa 70% of all leukocytes in decidual tissue. uNK cells differ from peripheral NK cells in the expression of their receptor repertoire and in the expression of some genes induced by the hormonal environment. The main function of uNK cells is to regulate maternal uterine vasculature remodeling (51). Therefore, it has been demonstrated in the murine system that uNK cells produce proangiogenic factors such as VEGF and growth factors and provide local IFN-γ for initiation of spiral artery formation (52–54). Their origin or expansion remains a matter of discussion. They may migrate from the periphery, differentiating from NK cell progenitors under the control of different factors, including steroid hormones (55), and/or by recruitment of peripheral NK cells into the uterus (56–58) or expand *in situ* after pregnancy was established (59). Qu and colleagues demonstrated a P4-dependent osteopontin expression in human decidual stroma cells and human uNK cells and proposed a role for osteopontin in uNK cell accumulation in uterine tissue (60). Moreover, human uNK cell recruitment from the peripheral blood into the uterus seems to be favored by rising E2 and LH levels and restricted by increasing amounts of P4 (61). Interestingly, mature human and rodent uNK cells do not express steroid receptors (55, 62–64). Thus, it is suggested that, at least for P4, effects are mediated through the glucocorticoid receptor (GR), proven to be expressed on murine uNK cells (65). In addition to the lack of steroid receptors, uNKs also miss the classical LH/CG receptor. Thus, hCG was suggested to induce human uNK cell proliferation through the mannose receptor (66). Regarding the function of uNK cells, they have been shown to contain high amounts of perforin but only display low cytotoxic activity. Several studies indicated that P4 and its mediator PIBF inhibit human NK cell activity via a block of degranulation (67–70). In agreement, E2 increased human and murine NK cell number but reduced their cytotoxicity (71, 72). By contrast Kitaya and colleagues as well as Kurashige and colleagues revealed that neither P4 nor E2 had significant effects on the proliferation, cytolytic activity, and cytokine secretion of human endometrial NK cells (73, 74). This was also true for AFP (73). In contrast to these results, it was shown that hCG and LH applied to virgin mice resulted in an enhanced NK cell activity (75, 76) suggesting that pregnancy hormones differently regulate NK cell function. Moreover, contradictory results between humans and mice regarding an influence of pregnancy hormones on different immune cell populations depict the limitations of animal models in understanding mechanisms unique to human pregnancy. Finally, hCG not only influences NK cells but is also produced and secreted by them during pregnancy (77). Hormonal effects on NK cells are summarized in Figures 1 and 2.

### Effect of pregnancy hormones on mast cells

Mast cells (MCs) are best known for their effector function in allergic diseases. After binding of allergen-specific IgE to its receptor (FcεRI), preformed and newly synthesized mediators stored within the MCs are released to induce inflammatory immune responses (78). Beside this well-documented function of MCs, recent data suggest MCs as critical regulators of adaptive immune responses (79). Additionally, we recently uncovered a critical role for MCs in pregnancy success. To study the importance of MCs in murine pregnancy, we took advantage of a MC-deficient mouse model. We found uterine MCs (uMCs) to have a unique phenotype. uMCs increase in number every time a female becomes receptive and rapidly expand after pregnancy occurs. In the absence of MCs implantation is severely impaired and spiral artery remodeling has shown to be insufficient. This is suggested to result in fetuses that are growth-retarded (80). We also proposed a role for uMCs in trophoblast survival, placentation, and fetal growth. MC salutary role in murine pregnancy is mediated at least in part by Galectin-1 (80). Oscillations in the number of uMCs during the reproductive cycle in humans (81) and mice (82) seem to be, at least partially, hormone regulated. Several studies demonstrated that P4 and E2 influence rat and mouse MC density in different tissues, including mammary glands and uterine tissue (82–85). We additionally suggested a function for both hormones in the recruitment of murine MCs from the periphery into the uterus as well as an impact on MC activity (86). In agreement, several studies confirmed a major effect of P4 and E2 on rat, mouse, and human MC activation (83, 87–90). By contrast, two other studies did not observe alterations in the number of granulated or degranulated rat and mouse MCs after P4 or E2 treatment (91, 92). Hunt and colleagues revealed that E2 influences the expression of iNOS and TNF-α in murine uMC (93). It can be assumed that the observed effects of P4 and E2 on MCs are in part of direct nature as MCs from different species have been proven to express P4 and E2 receptors (85–87, 89, 94). Studies analyzing the impact of hCG on MCs are rare to find in the literature. One study investigated the histamine content in MCs in a model of ovarian hyperstimulation induced in rabbits. However, hCG application seemed not to change the histamine content in MCs (95). Hormonal effects on MCs are summarized in Figures 1 and 2.

### Effect of pregnancy hormones on dendritic cells

As professional antigen-presenting cells, DCs are at the interphase between the innate and the adaptive immune system; hence their activation and modulation is critical for the outcome of the immune response. Dependent on their activation status, DCs either secrete pro- or anti-inflammatory cytokines, thereby inducing immune responses or suppressing them, respectively. During normal pregnancy, the majority of human and murine decidual DCs presents an immature (tolerogenic) phenotype and mainly produce IL-10, thus contributing to a fetus-friendly local environment (96, 97). In line with this, spontaneous abortions in humans and mice are associated with an increased number of mature, IL-12-producing DCs (98, 99). Moreover, the importance of DCs for a proper decidualization and implantation has been nicely shown by Plaks and colleagues in a mouse model (100). Data regarding the influence of pregnancy-associated hormones on DC function are widespread and inconsistent. DCs derived from bone-marrow precursors or monocytes as well as DCs from spleen or decidua have been shown to react differentially to hormonal stimulation. This may offer a possible explanation how the endocrine system supports the pregnant immune system in tolerating the semi-allogeneic fetus while at the same time being aware of pathogens; these cells being pleiotropic and it should not come as a surprise that they have the machinery to respond differently depending on the situation. DCs are highly susceptible to hormonal stimulation by expressing receptors for P4, E2, hCG, and LH (101–103). Hormonal stimulation of activated bone-marrow derived DCs (BMDCs) resulted in the majority of studies in an impaired up-regulation of MHCII molecules and co-stimulatory molecules associated with a reduced capability to secrete pro-inflammatory cytokines (101, 104). This would imply that upon hormonal stimulation they acquire a rather pregnancy-friendly phenotype. The concrete impact of P4 on IL-10 secretion by activated rat and mouse BMDCs was differentially discussed and has to be further evaluated (101, 104, 105). In line with the hormonal-mediated induction of a tolerogenic phenotype in activated BMDCs, their T-cell stimulatory capacity was reduced. This suggests that hormone-treated DCs have a pregnancy-protective effect by suppressing alloreactive T cell responses (101, 104, 105). However, results obtained after the addition of P4, E2, and hCG to monocyte-derived DCs (moDCs) from human peripheral blood are different from the results obtained after hormonal stimulation of BMDCs. Here, two studies showed that combinations of P4, E2, and hCG did not change the expression of maturation markers of moDCs or their T cell stimulatory capacity (106, 107). Segerer and colleagues demonstrated, at least for hCG, a pregnancy-positive effect on HLA-DR expression associated with a significant reduction in the ability to stimulate T cells (108). Of great interest is the fact that most of the published studies observed a significant up-regulation of IL-10 production by human DCs after treatment with pregnancy hormones (106, 107, 109). Uemura and colleagues additionally proposed an induction of T cell differentiation into Th2 (107), a phenotype that is reportedly pregnancy-friendly. Analysis of hormonal effects on splenic DCs has been performed in several mouse models, including models of autoimmune diseases and pregnancy. In a murine model for multiple sclerosis, namely experimental autoimmune encephalomyelitis (EAE), E2 treatment has shown to be protective. Here, E2 did not affect the expression of activation markers and co-stimulatory molecules of DCs but inhibit their ability to stimulate T cell proliferation and secretion of Th1 and Th2 cytokines. The reduced T cell stimulatory capacity was suggested to be due to an increased expression of indoleamine 2,3-dioxygenase (IDO) in DCs after E2 treatment (110, 111). Accordingly, hCG was also proven to up-regulate IDO in DCs in a murine model of autoimmune diabetes (112). As for E2, it was reported that P4 seems to affect the ratio of Th1-promoting DEC-205^+^ DCs and Th2-promoting 33D1^+^ DCs during pregnancy, favoring the dominance of 33D1^+^ DCs. The need of 33D1^+^ DCs for pregnancy success results from the observation that depletion of this specific DC subset during the perinatal period in mice caused substantial fetal loss probably mediated through Th1 up-regulation via transient IL-12 secretion (113). We have recently investigated the influence of hCG and LH on the number and phenotype of peripheral and local DCs in a murine model of disturbed tolerance to the semi-allogeneic fetus. We found that the *in vivo* application of both hormones prevented fetal rejection and this was associated with a reduced number of total and mature DCs both in the periphery and in decidua. Furthermore, we proved that hCG-treated decidual DCs had an elevated capacity to induce regulatory T (Treg) cells (103). This confirms the pregnancy-protective impact of both gonadotropins via modulation of adaptive immune responses. Effects of AFP on different DC subsets during pregnancy are almost unknown. Evidence for an AFP-mediated induction of tolerogenic DCs came from a tumor model where AFP has been demonstrated to induce tolerogenic DC capable of suppressing tumor-specific CD8^+^ cytotoxic T lymphocytes within the tumor (114). Hormonal effects on DCs are summarized in Figures 1 and 2.

### Effect of pregnancy hormones on B cells

**Figure 3:**
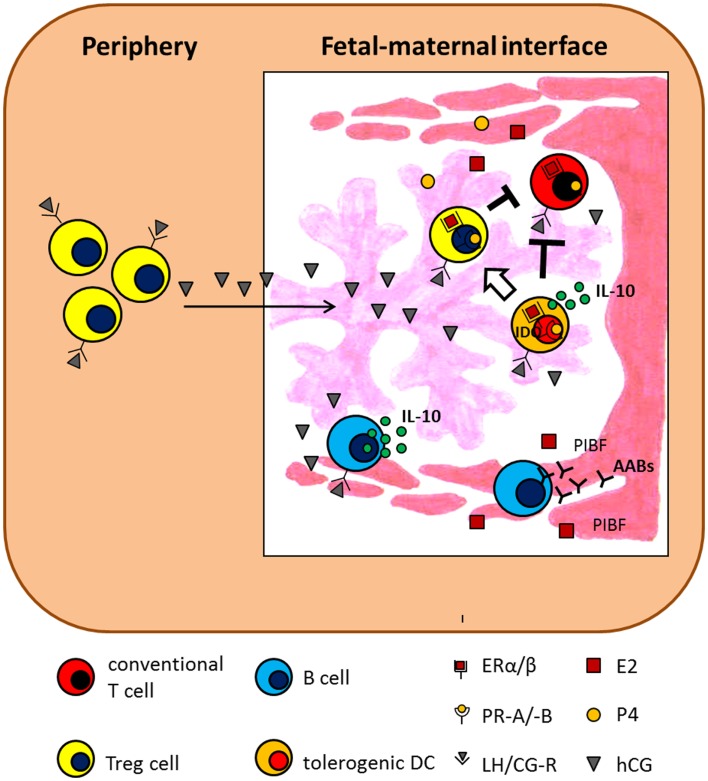
**Hypothetical scenario presenting the influence of pregnancy-associated hormones on adaptive immunity**. The scenario suggests several mechanisms by which E2, P4, and hCG influence adaptive immune cells and thereby support pregnancy success. AABs, asymmetric antibodies; ER, estrogen receptor; IDO, indoleamine 2,3-dioxygenase; IL-10, interleukin-10; LH/CG-R, luteinizing hormone/chorionic gonadotropin receptor; PIBF, progesterone-induced blocking factor; PR, progesterone receptor.

B lymphocytes are allocated to the adaptive immune system and they are best known for their capability to secrete antibodies. However, B cells do more than producing antibodies. They efficiently present antigens and modulate the function of T cells and DCs by producing cytokines (115, 116). During pregnancy, various B cell subsets are proposed to differently affect pregnancy outcome [reviewed in Ref. (117)]. Basically, B cells can be divided in two main populations, namely B1 and B2 B cells. These two populations differ in their developmental origin, surface marker expression and function (118). B1 B cells are then further subdivided in B1a and B1b B cells based on the expression of the surface marker CD5 (119). Interestingly, B1a B cells produce a specific type of antibody, the so-called natural antibodies (120). Due to their poly-reactive nature, natural antibodies are suggested to induce autoreactivity and thus are involved in the onset of autoimmune diseases (121). Even during pregnancy, B1a B cells producing natural antibodies are proposed to have detrimental effects on pregnancy outcome. We recently showed that the number of B1a B cells significantly decrease in the third trimester of normal pregnant women while remain elevated in pre-eclamptic patients (122). As patients suffering from pre-eclampsia have augmented serum hCG levels compared to normal pregnant women, we assumed that hCG may be responsible for the increased B1a B cell number. This assumption is further underlined by the fact that almost all B1a B cells express the LH/CG receptor and expand *in vitro* upon hCG stimulation (122). It was however shown that hCG inhibited antibody formation of murine B cells (123, 124). However, these two mentioned studies did not focus their analysis on B1a B cells but in total B cells. It is tempting to speculate that hCG may influence cell phenotype and antibody production differently in different B cell subsets. In contrast to the detrimental effect proposed for natural antibodies on pregnancy outcome, asymmetric antibodies (AABs), due to their structural anomaly, favor pregnancy success by reducing alloreactive immune responses (125). AABs increase during pregnancy and their lack is associated with pregnancy failure in humans (126, 127). Secretion of AABs seems to be, at least partially, hormone regulated. P4 but not E2 provoke AAB secretion (128). Hereby, P4 mediates its function via induction of PIBF (129). Beside an influence on antibody formation and secretion, pregnancy hormones also regulate B cell development and cytokine secretion. Administration of E2 alone, or in combination with P4, preferentially suppressed IL-7 responding cells and their progeny in bone-marrow (130). In contrast to P4 and E2, human B cell stimulation by hCG seems to be highly dependent on hCG doses and purity of hCG preparations (131–133). Recently, we proposed another pregnancy-protective effect of hCG, namely its capacity of increasing the regulatory function of human regulatory B cells (Breg or B10) shown to contribute to fetal survival. Breg express the LH/CG receptor and increase their IL-10 production in response to hCG treatment (134). Hormonal effects on B cells are summarized in Figure 3.

### Effect of pregnancy hormones on T cells

T cells are key regulators of both the antibody and the cell-mediated arms of the adaptive immune system and can be divided into two subcategories, CD4 expressing T helper cells and cytotoxic T cells expressing CD8. According to their cytokine secretion pattern, they are usually further subdivided in Th1, Th2, and Th3 cells although it is well known that this classification is oversimplified as further subsets as Th9 and Th17 has been described. T cells mediate their function either by direct cell–cell contact or indirectly by the secretion of cytokines defining the local environment as a pro-inflammatory or anti-inflammatory one. Normal pregnancy is associated with a pro-inflammatory Th1 profile at early and late pregnancy stages being important for a proper blastocyst implantation and initiation of labor, respectively. At midgestation, an anti-inflammatory Th2 profile guarantees tolerance of the foreign fetal antigens. Imbalances in cytokine profiles were associated with human pregnancy complications (135), suggesting a major part for T cells in fetal tolerance by regulating the local cytokine milieu. Steroid hormones are reportedly involved in modulating cytokine secretion by different T cell subsets. P4 and E2 are proposed to influence the Th1/Th2 balance favoring a Th2 predominance at the fetal–maternal interface in humans and mice (136–144). Hormonal effects are mainly mediated via their classical receptors expressed by human and murine T cells (16, 103, 143, 145, 146). However, Chien and colleagues provide evidence that P4 may act through non-classical steroid receptors to cause immune modulation and suppression of T cell activation during pregnancy (147). In addition to regulate the cytokine secretion of T cells, steroid hormones and their mediators (e.g., glycodelin A) induce apoptosis of effector T cells (148–150) and increase the expression of pregnancy-protective molecules such as the leukemia inhibitory factor (LIF) known to modulate immune responses during early human and murine pregnancy (151, 152). In line with this, hCG is proposed to support fetal survival by modulating murine T cell activity and function (153, 154). More precisely, Khil and colleagues demonstrated that hCG prevented the development of the disease in a murine model of autoimmune diabetes. Here, hCG treatment efficiently suppressed IFN-γ production, but increased IL-10 and TGF-β production in splenocytes. hCG application also suppressed TNF-α production (155). In contrast, AFP apparently has no direct effect on T lymphocytes. T cells stimulated with AFP retain their full capacity to respond in mixed lymphocytes culture (MLC) and cell-mediated lympholysis. However, AFP provokes major changes in the functional status of monocyte-enriched, MLC-stimulating cell population. This monocyte-enriched population induces T suppressor cells while at the same time, suppresses generation of cytotoxic T cells (156–158).

Pregnancy-associated hormones not only affect conventional T cells but have also been shown to support the generation and function of Treg cells. Treg cells represent a specialized subset within the T cell compartment with unique properties. During pregnancy, they are fundamentally involved in the suppression of alloreactive immune responses and, thus their absence results in worse pregnancy outcome both in humans (159) and mice (160). The presence of Treg cells in the uterus before pregnancy seems to be very relevant as well. A diminished endometrial expression of their major transcription factor Foxp3 is associated with infertility in women (161) while depletion of Treg cells in a murine model derived in a hostile uterine microenvironment hindered the implantation of the embryo (162). During the reproductive cycle, Treg cells fluctuate (162–164) and this was suggested to be hormone-driven (165). This could be interpreted as hormones preparing the mother and particularly the tissue for pregnancy. Already in the 80s hCG was assumed to induce human and murine suppressor T lymphocytes (166–168). Nowadays, steroid hormones and gonadotropins are proposed to regulate several aspects of Treg cell biology, including generation, expansion, migration, and suppressive function both in humans and mice (103, 145, 169–176). However, some studies provide evidence that P4 and E2 induce a reduction of Treg cells or did not provoke changes in the number of Treg cells (177–179). Thus, these contradictory findings may depend on the markers used to define Treg cells, the time point of analysis and the model used. Hence, more research is needed to understand at which time point and through, which mechanisms pregnancy hormones influence Treg cell function. Hormonal effects on T cells are summarized in Figures 2 and 3.

## Therapeutic Potential of Pregnancy-Associated Hormones in Assisted Reproductive Techniques

Immunological disorders have been suggested as one of the major reasons for unexplained infertility, implantation failures, and recurrent pregnancy loss. It has been discussed that alteration in the number and function of immune cell populations during the reproductive cycle and in early pregnancy stages may result in the inability to become pregnant and in worse pregnancy outcome. Women undergoing assisted reproductive techniques (ART) often present recurrent implantation failures after *in vitro* fertilization (IVF) and embryo transfer (ET). Hormonal treatment with steroid hormones and gonadotropins before and after ART was shown to support successful implantation and thereby improve pregnancy rates (26, 180–182). Based on the data discussed here, it can be speculated that hormonal treatment in ART counteract immunological disorders and may therefore, at least partially explain improved pregnancy rates. By affecting immune cell populations and their products, hormones are suggested to influence the local environment and thereby support an optimal implantation of the embryo (3). Lukassen and colleagues showed that hormonal stimulation for IVF treatment positively affected the CD56^bright^/CD56^dim^ ratio in the endometrium by a relative decrease in the cytotoxic CD56^dim^ CD16^+^ NK cell number. Moreover, the same authors observed an increase in B cells and macrophages and these effects were restricted to the endometrium and could not be observed in peripheral blood (183). By doing so, hormones may contribute to tissue remodeling and angiogenesis resulting in a proper placentation and fetal nourishment. The positive effect of hCG on ART is underlined by a study of Mansour and colleagues who showed that intrauterine injection of hCG before ET in patients undergoing IVF significantly improved implantation and pregnancy rates (180). Unfortunately, the authors did not analyze the number and activity of Treg cells although augmented pregnancy rates after IVF have been associated with elevated Treg cell numbers in peripheral blood (184). Interestingly, based on our observation that hCG efficiently attracts Treg cells to trophoblasts (145), the Egyptian IVF-ET Center conducted a clinical trial investigating the effect of intrauterine injection of hCG on endometrial Treg cells. Upcoming results will prove whether hCG treatment around implantation time may increase endogenous endometrial Treg cell levels and thereby favor the implantation process.

## Conclusion

Altogether, an exhaustive analysis of the literature indicates a crucial role for sex hormones on a variety of immune cell populations building up a complex network of interactions between the endocrine and the immune system. Irritations in this fine regulated balance may result in implantation failure and undesired pregnancy outcome. Thus, further investigations on the immune modulating functions of pregnancy-associated hormones will improve our understanding of endocrine–immune interactions before and during pregnancy and may help to develop selective strategies in the treatment of infertility and pregnancy complications.

## Conflict of Interest Statement

The authors declare that the research was conducted in the absence of any commercial or financial relationships that could be construed as a potential conflict of interest.
